# Safety and Effectiveness of Bupropion for Smoking Cessation During Pregnancy: A Scoping Review

**DOI:** 10.7759/cureus.118111

**Published:** 2026-10-18

**Authors:** George R Habib, Priyanka Ramanathan, Gabriella Garas, Alexander Knight

**Affiliations:** 1 Dr. Kiran C. Patel College of Osteopathic Medicine, Nova Southeastern University, Fort Lauderdale, USA; 2 Obstetrics and Gynecology, Alabama College of Osteopathic Medicine, Dothan, USA; 3 Psychiatry, Morton Plant North Bay Hospital Recovery Center, Lutz, USA

**Keywords:** bupropion, fetal outcomes, maternal safety, neonatal outcomes, nicotine dependence, pregnancy, psychopharmacology, scoping review, smoking cessation, tobacco use

## Abstract

Smoking during pregnancy is associated with adverse maternal, fetal, and neonatal outcomes. Although behavioral counseling remains first-line treatment, pharmacotherapy may be considered when counseling alone is insufficient. This scoping review mapped the available evidence on the safety and effectiveness of bupropion for smoking cessation during pregnancy.

The review was conducted according to Joanna Briggs Institute methodology and reported in accordance with the Preferred Reporting Items for Systematic Reviews and Meta-Analyses extension for Scoping Reviews (PRISMA-ScR). MEDLINE (PubMed), Embase, the Cochrane Library, PsycINFO, and ClinicalTrials.gov were searched without date restrictions for English-language studies evaluating bupropion for smoking cessation during pregnancy. Two reviewers independently screened records, and the review team extracted data on smoking cessation, maternal safety, and fetal or neonatal outcomes.

Of 599 records identified, 13 sources of evidence met the inclusion criteria. Maternal and fetal safety findings were generally reassuring, with no consistent increase in maternal adverse events, major congenital malformations, preterm birth, impaired fetal growth, or serious neonatal morbidity. Evidence for smoking cessation effectiveness was mixed. Some studies reported improved short-term abstinence, reduced cigarette consumption or tobacco exposure, and improvement in craving or withdrawal symptoms, whereas other randomized trials found no significant improvement in sustained abstinence compared with placebo or counseling. Interpretation was limited by small samples, few bupropion-exposed pregnancies, heterogeneous designs and outcomes, and restrictive trial eligibility criteria.

Current evidence is more reassuring regarding maternal, fetal, and neonatal safety than it is conclusive regarding smoking cessation effectiveness. Larger, adequately powered studies involving more representative pregnant populations are needed to clarify efficacy and detect uncommon adverse outcomes.

## Introduction and background

Sustained-release bupropion is an FDA-approved medication for smoking cessation in the general population and is also used as an antidepressant [1]. However, its role as a smoking cessation pharmacotherapy during pregnancy remains less clearly established. Current clinical guidance from the American College of Obstetricians and Gynecologists (ACOG) emphasizes individualized risk-benefit discussion when pharmacotherapy is considered during pregnancy rather than routine use for all pregnant smokers [2]. In the general population, bupropion improves smoking cessation outcomes, with users being 49-72% more likely to successfully stop smoking compared with placebo or no pharmacotherapy [3]. However, it remains unclear whether this benefit extends to pregnant patients.

Smoking during pregnancy is associated with adverse maternal, fetal, and neonatal outcomes, most notably low birth weight. Many women who are able to stop smoking do so by the first prenatal visit, while those who continue smoking beyond that point are more likely to continue throughout pregnancy without additional support [4]. Behavioral counseling remains the foundation of smoking cessation treatment during pregnancy, but counseling alone may not be sufficient for all patients, particularly those with higher nicotine dependence, psychosocial stressors, or co-occurring psychiatric symptoms.

Bupropion occupies a distinct clinical role because it is prescribed both as an antidepressant and as a pharmacologic aid for smoking cessation. In pregnancy, this overlap is especially relevant because nicotine dependence may coexist with depression, anxiety, or other psychiatric symptoms. Clinicians considering bupropion must therefore weigh the risks of continued tobacco exposure, untreated or undertreated psychiatric illness, and fetal medication exposure together. This decision is further complicated by bupropion's known safety considerations, including dose-related seizure risk, contraindications in patients with seizure disorders or other factors that lower the seizure threshold, and labeling warnings regarding suicidal thoughts and behaviors with antidepressant use and neuropsychiatric adverse events reported during smoking cessation treatment [1].

When bupropion is considered specifically for smoking cessation during pregnancy, both effectiveness and safety are important. Existing pregnancy data have generally been reassuring regarding major congenital malformations, but the evidence remains limited by small numbers of exposed pregnancies and difficulty separating bupropion use for smoking cessation from use for depression or other psychiatric indications [5-7]. Previous reviews have evaluated pharmacologic smoking cessation interventions during pregnancy broadly or have focused primarily on the gestational safety of bupropion and varenicline [8,9]. However, the evidence base has expanded with additional randomized and population-based studies, and the literature has not been recently mapped with a bupropion-specific focus that integrates smoking cessation effectiveness with maternal, fetal, and neonatal safety outcomes.

This scoping review aims to map the available literature on bupropion as a smoking cessation pharmacotherapy during pregnancy. Specifically, it summarizes the current evidence regarding smoking cessation effectiveness, maternal safety and tolerability, and fetal and neonatal outcomes.

## Review

Review question

What is the existing evidence regarding the safety and effectiveness of bupropion for smoking cessation during pregnancy?

Inclusion criteria

Participants

This review included studies involving pregnant women at any gestational age who used any nicotine products. Studies were eligible when nicotine use was documented, regardless of product type or frequency of use. Studies including mixed substance use populations were included if the outcomes for nicotine-using pregnant participants receiving bupropion could be identified. No restrictions were placed on age, parity, comorbid conditions, or race/ethnicity.

Concept

This review evaluated the use of bupropion as a pharmacologic therapy for smoking cessation in pregnant women. This included any formulation or dosage of bupropion that was used with the intention of reducing or stopping nicotine use. Studies in which bupropion exposure was not restricted exclusively to smoking cessation were included when relevant safety outcomes could inform clinical decision-making about bupropion use in pregnant smokers; however, indication ambiguity was recorded and considered during interpretation. This review considered any maternal smoking-cessation outcomes (cessation in use, reduction in use, relapse rates, treatment adherence, etc.), maternal safety outcomes, and fetal and neonatal outcomes (including congenital anomalies and poor fetal outcomes).

Included studies could use any type of comparison agent such as NRT (nicotine replacement therapy), other pharmacotherapies, behavioral interventions, and placebos. This review incorporated evidence from studies that examined and reported bupropion use for smoking cessation during pregnancy regardless of coadministration with other agents or combinations with behavioral therapy.

Context

This review included studies conducted in any clinical or community setting in which data were available on bupropion use for smoking cessation in pregnant women. This included outpatient primary care settings, prenatal care clinics, obstetrical clinics, addiction clinics, or community health settings. No restrictions were placed on healthcare settings, geographical location, or cultural context.

Source Classifications

This scoping review considered any type of published primary empirical study designs including randomized controlled trials, quasi-experimental studies, observational studies, prospective and retrospective cohort studies, case-control studies, cross-sectional studies, case series, and individual case reports.

No restrictions on publication date were applied. Studies from the inception of the database to the date of search were eligible. Only studies available in English were included due to resource limitations.

Review articles such as meta-analyses, literature reviews, or narrative reviews were excluded as sources of evidence, but their reference lists were screened to identify additional primary sources. This was to prevent double reporting of data. Clinical guidelines given by organizations were also excluded unless they contained original primary data. Unpublished sources without available primary data were not considered. Conference abstracts and clinical trial registries were only considered if they provided extractable data relevant to the review question. Studies conducted with animal models or in vitro settings were excluded.

Methods

This scoping review was conducted in accordance with the Joanna Briggs Institute (JBI) methodology for scoping reviews [10]. The review was reported in accordance with the Preferred Reporting Items for Systematic Reviews and Meta-Analyses extension for Scoping Reviews (PRISMA-ScR) guidelines [11]. A review protocol was developed before study selection but was not prospectively registered.

Search Strategy

The search strategy aimed to locate published primary empirical studies and registered studies with available results relevant to the use of bupropion for smoking cessation during pregnancy. A three-step search strategy was employed in accordance with JBI guidance.

First, an initial limited search of MEDLINE (via PubMed) and Embase was conducted to identify relevant articles. The text words contained in the titles and abstracts of these articles, along with the controlled vocabulary terms used to describe them, were analyzed and used to develop a comprehensive search strategy. Next, the full search strategy, including all identified keywords and index terms, was adapted and applied to all included databases. The databases searched included MEDLINE (via PubMed), Embase, the Cochrane Library, and PsycINFO. ClinicalTrials.gov was also searched to identify relevant studies with available results. The search was limited to studies published in English. No restrictions on publication date were applied. All searches were conducted from database inception through 02/14/2026. Finally, the reference lists of relevant review articles were screened to identify additional eligible primary sources. A full search strategy is available in the Appendices.

Study/Source of Evidence Selection

After the search, all identified citations were collected and imported into reference management software and Rayyan (Rayyan Systems Inc., Cambridge, MA, USA) for deduplication and screening [12]. Titles and abstracts were screened by two independent reviewers for assessment against the inclusion criteria for the review. Studies that appear to meet the criteria or were unclear in their eligibility were retrieved in full text. The full-text articles were independently assessed by two reviewers to determine the final eligibility. Disagreements that arose at any stage of the screening process were resolved with consultation of a third reviewer when a consensus could not be reached. Reasons for the exclusion of all removed studies and the selection process were documented and presented using a PRISMA 2020 flow diagram shown in Figure 1 [13].

**Figure 1:**
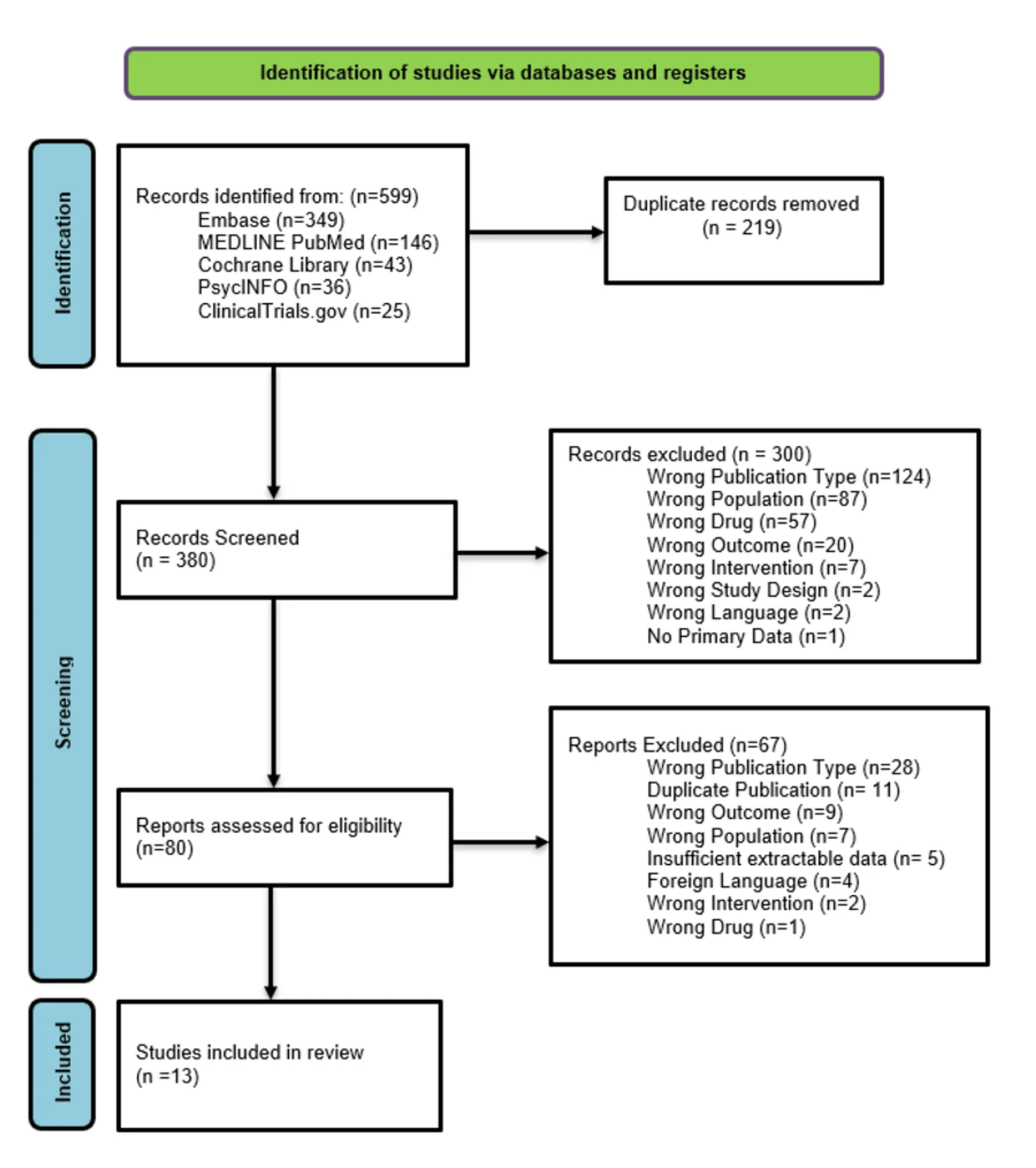
PRISMA 2020 flow diagram of study selection PRISMA: Preferred Reporting Items for Systematic Reviews and Meta-Analyses

Data Extraction

Data were extracted from the included studies using a summary table developed by the review team. Three reviewers independently extracted data from each included study. Extracted data included study identifiers (e.g., DOIs), titles, study designs, participant characteristics including number of bupropion users, reported maternal smoking-cessation outcomes, maternal safety outcomes, and fetal or neonatal outcomes, and key limitations of the study. Any disagreements during data extraction were resolved through discussion. Funding sources for the included sources of evidence were extracted when reported and summarized narratively.

Data Analysis and Presentation

Extracted data were summarized descriptively and presented in table and narrative formats. Tables were used to summarize key study characteristics, including study design, population of bupropion users, and reported maternal and fetal or neonatal outcomes. The results were mapped to describe the extent of the available evidence on bupropion use for smoking cessation during pregnancy, the efficacy for smoking cessation, and the safety of its use. No formal critical appraisal of individual sources of evidence was performed in accordance with JBI guidance for scoping reviews.

Results

Study Selection and Characteristics

The search identified 599 records, of which 13 met the inclusion criteria after title and abstract screening and full-text review. The evidence base comprised four randomized controlled trials, eight cohort or observational studies, and one case-based clinical report. The included studies evaluated smoking cessation effectiveness, maternal safety and tolerability, and fetal or neonatal outcomes. Sample sizes and study designs varied considerably, ranging from small pilot trials and observational studies to large population-based cohorts. Funding sources varied across the included studies and included governmental, institutional, and nonprofit support. Boshier et al. [14] reported that the Drug Safety Research Unit received a non-contractual donation from the company marketing bupropion, with no funder involvement in study conduct or conclusions. Several studies did not report a specific funding source. Characteristics and key findings of the included sources are summarized in Table 1.

**Table 1:** Characteristics and key findings of the included sources of evidence aRR: adjusted risk ratio; CI: confidence interval; HR: hazard ratio; ITT: intention-to-treat; RCT: randomized controlled trial

Study/DOI	Design and sample	Number of participants using bupropion	Smoking cessation findings	Maternal, fetal, and neonatal safety findings	Key limitations
Miller et al. [15]/10.1016/j.ajog.2003.10.260	Pilot RCT; 76 participants, 33 assigned bupropion	33	Greater smoking reduction at 4 weeks with bupropion (ITT: 50% vs 18%; p=0.008); no significant abstinence advantage and no sustained reduction at 8 weeks	Maternal and fetal safety outcomes were not reported	Conference abstract only; limited methodological detail and no full safety reporting
Boshier et al. [14]/10.1007/s00228-003-0693-0	Post-marketing observational cohort; 12 first-trimester bupropion-exposed pregnancies, with outcomes ascertained in 8	12	Not evaluated	Outcomes were ascertained in eight pregnancies: five live births, two therapeutic terminations, and one intrauterine death; no congenital anomalies were reported among live births	Very small pregnancy subgroup within post-marketing surveillance; no comparator group
Chan et al. [16]/10.1300/J069v24n02_02	Prospective matched controlled observational study; 22 bupropion-exposed participants and 22 matched controls	22	Smoking cessation occurred in 45% of the bupropion group versus 14% of controls (p=0.047); cigarette reduction among continuing smokers was similar between groups	All participants delivered healthy, full-term infants	Small, nonrandomized sample; residual confounding and baseline differences in prior quit attempts remain possible
Chun-Fai-Chan et al. [5]/10.1016/j.ajog.2004.09.027	Prospective comparative cohort; 136 bupropion-exposed pregnancies	136	Not evaluated	No major congenital malformations were observed, and the study did not suggest an increased risk above baseline. Spontaneous abortions were significantly more frequent than in the nonteratogen comparison group (14.7% vs 4.5%; p=0.009) but occurred at rates similar to those reported in previous studies of other antidepressants	Exposure indication was not restricted to smoking cessation; observational design
Chisolm et al. [17]/10.3109/00952990903544844	Retrospective cohort within a substance-use treatment program; 503 patients overall, including 11 bupropion users	11	Mean cigarette reduction was 6.4 cigarettes/day with bupropion versus 0.4 with citalopram or escitalopram; the difference was not significant (p=0.276)	No serious maternal adverse events were reported; fetal outcomes were not reported	Very small bupropion subgroup; retrospective records and potential confounding by treatment selection and co-occurring substance use
Lundquist et al. [18]/10.1080/15504263.2012.671119	Case-based clinical report; 1 patient	1	Complete cessation was achieved during a combined intervention of bupropion, counseling, social support, and residential treatment	No original comparative safety data were reported; safety discussion was based on prior literature	Single case with multiple concurrent interventions; the independent effect of bupropion cannot be determined
Stotts et al. [19]/10.1055/s-0034-1386635	Double-blind, placebo-controlled pilot RCT; 11 enrolled (5 bupropion, 6 placebo) after 820 were screened	5	No bupropion participant achieved end-of-treatment 7-day abstinence; 2 placebo participants did. The sample was too small for efficacy inference	Adverse effects were mild and caused no discontinuations. Two serious adverse events in the bupropion group were judged unrelated; no fetal deaths, congenital anomalies, or births before 34 weeks were reported	Severe recruitment constraints and very small sample; psychiatric disorders or psychiatric medication use frequently led to exclusion
Bérard et al. [20]/10.1016/j.ajog.2016.06.059	Longitudinal cohort; 72 bupropion users	72	Smoking cessation occurred in 81% of bupropion users; 60% remained abstinent after treatment ended	Bupropion was associated with a lower risk of prematurity compared with continued smoking and was not associated with increased prematurity compared with nicotine patch replacement therapy	Observational design; treatment selection and adherence may confound cessation estimates
Nanovskaya et al. [21]/10.1016/j.ajog.2016.11.1036	Randomized, double-blind, placebo-controlled pilot RCT; 65 total (30 bupropion, 35 placebo), with counseling in both groups	30	Higher abstinence during treatment (19% vs 2%; p=0.003) and lower urinary cotinine (348 vs 831 ng/mL; p=0.007); no significant abstinence difference at treatment end or the end of pregnancy	No significant differences in maternal measures, neonatal anthropometrics, 5-minute Apgar score, or cord blood pH; serious adverse events did not differ significantly between groups	Small sample and high attrition; limited power for sustained abstinence and uncommon safety outcomes
Tran et al. [22]/10.1186/s12916-019-1472-9	Population-based retrospective cohort; 233 bupropion-exposed pregnancies	233	Not evaluated	Composite adverse perinatal outcomes were similar to unexposed smokers (39.2% vs 39.3%; HR: 0.93; 95% CI: 0.73-1.19); no significant differences in small-for-gestational-age birth, special care admission, or severe neonatal morbidity	Limited bupropion exposure; insufficient power for bupropion-specific congenital anomaly analysis or comparisons across pharmacotherapies
Kranzler et al. [23]/10.1016/j.ajogmf.2021.100315	Multisite phase II placebo-controlled RCT; 129 total, with 64 assigned bupropion	64	No efficacy advantage: 7-day abstinence was 11% vs 18.5% at treatment end and 9.4% vs 21.5% at week 24 (bupropion vs placebo)	Treatment-related adverse events and maternal, pregnancy, and neonatal outcomes were comparable between groups	Not powered to assess rare safety outcomes; findings reflect second-trimester initiation under trial conditions
Tran et al. [24]/10.1001/jamainternmed.2025.0290	Multinational population-based retrospective cohort; 391,474 births, including 1,042 first-trimester bupropion exposures	1,042	Not evaluated	No increased risk of major congenital malformations overall compared with smoking without pharmacotherapy (aRR: 0.93; 95% CI: 0.67-1.29)	Too few exposed cases for robust analyses of specific malformation subgroups
Robijn et al. [25]/10.1111/add.70290	Multinational population-based retrospective cohort; bupropion-exposed pregnancies were too few for robust analysis	Too few for robust analysis	Bupropion exposure was insufficient for meaningful effectiveness estimates. Indication could not be isolated in Norway and Sweden because bupropion is also used for depression	Not a primary safety analysis	Low exposure prevalence, treatment nonadherence, potential residual confounding, and indication ambiguity in some countries

Maternal Safety and Tolerability

Across the studies reporting maternal safety outcomes, bupropion was generally well tolerated, and no consistent increase in maternal adverse events was identified. In a population-based cohort study, the risk of a composite made up of 10 maternal and neonatal outcomes did not differ significantly between bupropion-exposed and bupropion-unexposed pregnancies (39.2% vs 39.3%; HR: 0.93; 95% CI: 0.73-1.19). A nonsignificant increase in emergency cesarean delivery was observed among bupropion-exposed pregnancies [22].

The randomized trials similarly identified no significant maternal safety differences between treatment groups. Nanovskaya et al. [21] found no significant differences between bupropion and placebo groups in maternal blood pressure, pulse rate, or body mass index at the end of pregnancy. Headache, insomnia, dry mouth, and anxiety occurred in both groups without significant differences in frequency. One participant receiving bupropion developed superimposed preeclampsia requiring preterm delivery, although the event was considered unlikely to be related to the study medication.

In the smaller pilot trial by Stotts et al. [19], bupropion was generally well tolerated. Reported adverse effects included vomiting, dry mouth, and loss of appetite, none of which resulted in treatment discontinuation. Similarly, the larger randomized trial by Kranzler et al. [23] found no significant differences in treatment-related adverse events or other safety measures between the bupropion and placebo groups. The case-based clinical report by Lundquist et al. [18] did not provide comparative maternal safety data but characterized the available evidence regarding bupropion exposure in pregnancy as limited.

Fetal and Neonatal Outcomes

The available evidence did not demonstrate a consistent association between bupropion exposure and adverse fetal or neonatal outcomes. In the largest multinational study of congenital malformations, first-trimester bupropion exposure was not associated with an increased risk of major congenital malformations overall compared with smoking without pharmacotherapy (aRR: 0.93; 95% CI: 0.67-1.29). However, the number of bupropion-exposed pregnancies was insufficient for robust analysis of individual malformation subgroups [24].

Earlier observational evidence was generally consistent with this finding. In a prospective comparative study of 136 bupropion-exposed pregnancies, Chun-Fai-Chan et al. [5] reported no increase in the rate of major congenital malformations above the expected population baseline. Although bupropion exposure in this study was not restricted to smoking cessation, the study included participants using bupropion for smoking cessation and therefore contributed indirect pregnancy safety evidence relevant to clinical decision-making about bupropion use in pregnant smokers. The study did report a higher frequency of spontaneous abortions compared with the nonteratogen comparison group, although the authors noted that the rate was similar to that reported in studies of other antidepressants. Boshier et al. [14] similarly reported no congenital abnormalities among the small number of prospectively observed pregnancy exposures, although the number of exposed pregnancies was limited.

Population-based evidence also did not identify significant differences between bupropion-exposed and bupropion-unexposed pregnancies in preterm birth, small-for-gestational-age birth, neonatal special care admission, or severe neonatal morbidity [22]. Congenital malformations could not be evaluated specifically for bupropion in that study because of the limited number of exposed pregnancies.

The randomized trials were also generally consistent with the observational safety findings. Nanovskaya et al. [21] reported no significant differences between bupropion and placebo groups in birth weight, infant length, head circumference, five-minute Apgar score, or umbilical cord blood pH. Serious adverse events occurred in five participants in the placebo group and two in the bupropion group, and none were attributed to the study medication. Preterm birth before 34 weeks was uncommon and occurred at similar frequencies between groups.

Stotts et al. [19] reported no intrauterine fetal deaths, congenital anomalies, or births before 34 weeks in either treatment group. Mean birth weight was numerically higher among infants in the bupropion group than among those in the placebo group, although the small sample precluded meaningful statistical interpretation. Two neonatal serious adverse events occurred in the bupropion group and were determined to be unrelated to treatment.

In the larger randomized trial by Kranzler et al. [23], fetal and neonatal outcomes were also comparable between treatment groups. No significant differences were observed in gestational age at delivery, overall or spontaneous preterm birth, birth weight, size for gestational age, head circumference, or five-minute Apgar scores. The prospective matched observational study by Chan et al. [16] similarly reported that all 22 bupropion-exposed participants and 22 matched controls delivered healthy, full-term infants. The case-based report by Lundquist et al. [18] did not contribute original comparative fetal safety data and was therefore not used to characterize comparative fetal or neonatal risk.

Smoking Cessation Effectiveness

Evidence regarding the effectiveness of bupropion for smoking cessation during pregnancy was mixed. The randomized trials did not demonstrate a consistent effect on sustained abstinence, although some studies identified improvements in short-term abstinence, smoking reduction, or biomarkers of tobacco exposure.

In a randomized, placebo-controlled pilot trial, Nanovskaya et al. [21] found higher abstinence during the treatment period with bupropion than with placebo (19% vs 2%; p=0.003) and lower urinary cotinine concentrations (348 vs 831 ng/mL; p=0.007). Bupropion was also associated with reductions in cigarette cravings and nicotine withdrawal symptoms. However, abstinence rates did not differ significantly between groups at the end of treatment or at the end of pregnancy.

The pilot trial reported by Miller et al. [15] similarly found evidence of short-term smoking reduction without a significant improvement in abstinence. At four weeks, smoking reduction was more frequent in the bupropion group than in the control group in both the intention-to-treat analysis (50% vs 18%; p=0.008) and the available data analysis (67% vs 18%; p=0.008). Differences in abstinence were not statistically significant, and the reduction in smoking was not sustained at eight weeks.

The small pilot trial by Stotts et al. [19] was unable to provide meaningful estimates of cessation effectiveness because only 11 of 820 screened participants were enrolled. No participants receiving bupropion achieved biochemically confirmed seven-day abstinence at the end of treatment, compared with two participants receiving placebo. Among screened smokers who were excluded, psychiatric disorder was the most frequently reported exclusion reason (23%).

The largest randomized trial did not demonstrate an efficacy benefit. Kranzler et al. [23] reported seven-day point-prevalence abstinence rates of 11% with bupropion and 18.5% with placebo at the end of treatment and 9.4% and 21.5%, respectively, at week 24. Bupropion also did not significantly improve prolonged abstinence or reduce smoking among participants who continued to smoke. The trial included 129 pregnant smokers receiving bupropion or placebo in addition to smoking cessation counseling.

Observational studies reported more favorable cessation outcomes, although their findings varied. In the Quebec Pregnancy Cohort, 81% of the 72 bupropion users stopped smoking during pregnancy, and 60% remained abstinent after discontinuing pharmacotherapy [20]. In a smaller prospective matched study, smoking cessation occurred in 10 of 22 bupropion-exposed participants (45%) compared with three of 22 matched controls (14%; p=0.047) [16].

Among pregnant women receiving treatment for substance use disorders, Chisolm et al. [17] identified 11 bupropion users. The mean reduction in cigarettes smoked per day was greater with bupropion than with citalopram or escitalopram (6.4 vs 0.4 cigarettes per day), but the difference was not statistically significant (p=0.276). Smoking reduction in the bupropion group was also similar to that observed among participants who were not receiving antidepressant treatment.

Evidence from large population-based effectiveness studies remained limited by the small number of pregnancies exposed to bupropion. Robijn et al. [25] reported that bupropion exposure was too uncommon to support a meaningful effectiveness analysis across the participating countries. Data from Norway and Sweden were not included in the bupropion analysis because indication could not be reliably distinguished between smoking cessation and depression treatment. Tran et al. [22] primarily evaluated pregnancy safety and did not report bupropion-specific smoking cessation outcomes.

Finally, Lundquist et al. [18] described successful smoking cessation in a single patient receiving bupropion as part of a broader multidisciplinary intervention that also incorporated counseling, social support, and residential treatment. Given the case-based design and concurrent interventions, the independent contribution of bupropion to cessation could not be determined.

Discussion

This scoping review identified a limited and heterogeneous evidence base regarding bupropion for smoking cessation during pregnancy. The available literature was more consistent regarding safety than effectiveness. Across randomized and observational studies, bupropion was generally well tolerated, and no consistent increase in adverse maternal, fetal, or neonatal outcomes was identified. In contrast, evidence for smoking cessation effectiveness was mixed. Some studies reported short-term improvements in abstinence, cigarette reduction, or biomarkers of tobacco exposure, whereas others, including the largest randomized trial, did not demonstrate a sustained cessation benefit. These findings suggest that the evidence is generally reassuring regarding the absence of a clear safety signal but remains insufficient to establish consistent effectiveness for smoking cessation during pregnancy.

Maternal and fetal safety findings were broadly similar across different study designs. Randomized trials reported comparable adverse-event profiles between bupropion and placebo, while observational studies did not identify consistent increases in adverse pregnancy or neonatal outcomes [21-23]. Studies evaluating congenital malformations similarly did not demonstrate an increased risk of major congenital malformations overall [5,24]. However, these findings should be interpreted cautiously. Even large population-based studies included relatively few bupropion-exposed pregnancies, limiting the ability to evaluate rare outcomes or specific malformation subgroups. Therefore, the absence of a consistent safety signal is reassuring but should not be interpreted as definitive evidence that uncommon risks are absent.

Evidence regarding smoking cessation effectiveness was less consistent. Nanovskaya et al. [21] reported improvements in abstinence during active treatment, tobacco-exposure biomarkers, cravings, and withdrawal symptoms, but significant differences in abstinence were not maintained through the end of pregnancy. Miller et al. [15] similarly reported short-term smoking reduction without a sustained improvement in abstinence. In contrast, Kranzler et al. [23] found no significant cessation benefit of bupropion compared with placebo when both groups received smoking cessation counseling. Observational studies reported more favorable outcomes, including higher cessation rates in some bupropion-exposed groups [16,20]. However, differences in study design, participant selection, adherence, behavioral support, cessation definitions, and follow-up periods limit direct comparisons between studies. These findings suggest that bupropion may affect some intermediate measures of smoking behavior, but current evidence does not demonstrate a consistent effect on sustained abstinence.

An important limitation of the existing evidence is the limited representation of pregnant smokers with psychiatric comorbidity. In the pilot trial by Stotts et al. [19], only 11 of 820 screened individuals were ultimately enrolled, with psychiatric disorders and psychiatric medication use contributing substantially to exclusion. Such restrictions may limit the generalizability of trial findings to clinical populations in which smoking and psychiatric symptoms frequently coexist. This issue is particularly relevant to bupropion because of its dual roles as an antidepressant and smoking cessation medication. At the same time, this dual indication creates challenges for observational research, as demonstrated by difficulties distinguishing bupropion prescribed for smoking cessation from bupropion prescribed for depression in population-based datasets. Future studies should therefore more clearly document treatment indication, psychiatric comorbidities, concomitant psychotropic medication use, treatment adherence, and behavioral smoking cessation interventions.

These findings have implications for clinical decision-making but do not establish bupropion as a standard smoking cessation treatment during pregnancy. The available evidence does not demonstrate a consistent cessation benefit, yet it also does not identify a consistent pattern of maternal or fetal harm. Clinical decisions therefore remain dependent on individualized assessment of potential benefits and risks, particularly when smoking continues despite behavioral interventions. This uncertainty may be especially relevant when smoking cessation needs coexist with psychiatric treatment considerations. The available evidence also suggests that future research should move beyond highly selected trial populations and evaluate more representative groups of pregnant smokers, including those with psychiatric comorbidities who are commonly encountered in clinical practice.

This review has several limitations. The evidence base included heterogeneous study designs, populations, treatment protocols, and outcome definitions, which limited direct comparison and precluded quantitative synthesis. Several studies had small bupropion-exposed samples, while larger observational studies were limited by treatment-indication uncertainty and the small number of exposures available for specific analyses. Some safety evidence came from studies in which bupropion exposure was not restricted exclusively to smoking cessation. These studies were included because they contained pregnancy safety data relevant to bupropion exposure in pregnant smokers, but indication ambiguity limits direct application of their findings to smoking-cessation treatment decisions. No formal critical appraisal was performed, consistent with the scoping review methodology; therefore, this review maps the extent and direction of the evidence rather than establishing certainty of treatment effects. The restriction to English-language publications may also have excluded relevant evidence. Future research should include adequately powered multicenter studies with standardized cessation outcomes, longer follow-up, biochemical confirmation of smoking status when feasible, and clearer reporting of psychiatric comorbidity, treatment indication, adherence, and concurrent behavioral interventions.

## Conclusions

The available evidence regarding bupropion use for smoking cessation during pregnancy is more reassuring with respect to maternal, fetal, and neonatal safety than it is conclusive regarding smoking cessation effectiveness. Across the included studies, no consistent pattern of adverse pregnancy or neonatal outcomes was identified, whereas findings for abstinence and smoking reduction were mixed. Interpretation is limited by small randomized trials, heterogeneous outcome measures, limited bupropion exposure in population-based studies, and the underrepresentation of pregnant smokers with psychiatric comorbidities. These findings support individualized assessment of potential benefits and risks when pharmacotherapy is considered during pregnancy but do not establish bupropion as an effective standard treatment for smoking cessation in this population. Larger, adequately powered studies involving more representative clinical populations are needed to clarify both effectiveness and uncommon safety outcomes.

## Appendices

Complete search strategy

MEDLINE via PubMed

Date searched: 02/14/2026

Limits: English; no date restriction

Results: 146

Search strategy:

("Bupropion"[MeSH] OR bupropion[tiab] OR Zyban[tiab] OR Wellbutrin[tiab] OR Wellbutrin SR[tiab] OR Wellbutrin XL[tiab])

AND

("Smoking Cessation"[MeSH] OR "Tobacco Use Cessation"[MeSH] OR "Nicotine Dependence"[MeSH] OR smoking cessation[tiab] OR tobacco cessation[tiab] OR smoking abstinence[tiab] OR smoking reduction[tiab] OR tobacco reduction[tiab] OR quit*[tiab] OR abstinen*[tiab] OR cessation[tiab] OR nicotine dependence[tiab] OR tobacco use disorder[tiab])

AND

("Pregnancy"[MeSH] OR pregnan*[tiab] OR pregnant women[tiab] OR antenatal[tiab] OR prenatal[tiab] OR perinatal[tiab] OR gestation[tiab] OR maternal[tiab])

Embase

Date searched: 02/14/2026

Limits: English; no date restriction

Results: 349

Search strategy:

('bupropion'/exp OR bupropion:ti,ab,kw OR zyban:ti,ab,kw OR wellbutrin:ti,ab,kw OR 'wellbutrin sr':ti,ab,kw OR 'wellbutrin xl':ti,ab,kw) AND (((((('smoking cessation'/exp OR 'tobacco use cessation'/exp OR 'smoking reduction'/exp OR smoking) AND cessation:ti,ab,kw OR tobacco) AND cessation:ti,ab,kw OR smoking) AND abstinence:ti,ab,kw OR smoking) AND reduction:ti,ab,kw OR tobacco) AND reduction:ti,ab,kw OR quit*:ti,ab,kw OR cessation:ti,ab,kw OR abstinen*:ti,ab,kw OR 'nicotine dependence treatment':ti,ab,kw OR 'tobacco use disorder':ti,ab,kw OR 'nicotine dependence'/exp) AND ('pregnancy'/exp OR pregnan*:ti,ab,kw OR 'pregnant women':ti,ab,kw OR antenatal:ti,ab,kw OR prenatal:ti,ab,kw OR perinatal:ti,ab,kw OR gestation:ti,ab,kw OR maternal:ti,ab,kw)

Cochrane Library

Date searched: 02/14/2026

Limits: English; no date restriction

Results: 43

Search strategy:

(bupropion OR zyban OR wellbutrin OR "wellbutrin sr" OR "wellbutrin xl")

AND

("smoking cessation" OR "tobacco use cessation" OR "tobacco cessation" OR "smoking abstinence" OR "smoking reduction" OR "tobacco reduction" OR quit OR quitting OR abstinence OR "nicotine dependence" OR "nicotine dependence treatment" OR "tobacco use disorder")

AND

(pregnancy OR pregnant OR prenatal OR antenatal OR perinatal OR gestation OR maternal)

PsycINFO

Date searched: 02/14/2026

Limits: English; no date restriction

Results: 36

Search strategy:

(DE "Bupropion" OR bupropion OR Zyban OR Wellbutrin OR "Wellbutrin SR" OR "Wellbutrin XL")

AND

(DE "Smoking Cessation" OR DE "Nicotine Dependence" OR DE "Tobacco Smoking" OR "smoking cessation" OR "tobacco cessation" OR "smoking abstinence" OR "smoking reduction" OR "smoking behavior" OR "tobacco use" OR quit* OR abstinen* OR relapse prevention OR "nicotine dependence" OR "tobacco use disorder" OR "behavior therapy" OR counseling OR "motivational interviewing")

AND

(DE "Pregnancy" OR pregnan* OR prenatal OR antenatal OR perinatal OR maternal OR postpartum)

AND

(DE "Smoking Cessation" OR DE "Nicotine Dependence" OR DE "Tobacco Smoking" OR "smoking cessation" OR "tobacco cessation" OR "smoking abstinence" OR "smoking reduction" OR "smoking behavior" OR "tobacco use" OR quit* OR abstinen* OR relapse prevention OR "nicotine dependence" OR "tobacco use disorder" OR "behavior therapy" OR counseling OR "motivational interviewing")

AND

(DE "Pregnancy" OR pregnan* OR prenatal OR antenatal OR perinatal OR maternal OR postpartum)

ClinicalTrials.gov

Date searched: 02/14/2026

Limits: English; no date restriction

Results: 25

Search strategy:

(bupropion OR zyban OR wellbutrin) AND pregnancy
